# Three paradoxical paradigms of measles virus

**DOI:** 10.1371/journal.ppat.1014135

**Published:** 2026-04-15

**Authors:** Stephanie E. Clark, Patrick L. Sinn

**Affiliations:** 1 Microbiology and Immunology, Carver College of Medicine, The University of Iowa, Iowa City, Iowa, United States of America; 2 Stead Family Department of Pediatrics, Carver College of Medicine, The University of Iowa, Iowa City, Iowa, United States of America; Washington University School of Medicine in Saint Louis: Washington University in St Louis School of Medicine, UNITED STATES OF AMERICA

## Introduction

Measles is estimated to have diverged from rinderpest, a devastating cattle pathogen, around the sixth century BCE [1]. This is thought to have coincided with the rise of large cities, allowing for populations large enough (~250,000–500,000 people) to support continuous measles virus transmission. Measles today is highly adapted to humans and despite its zoonotic origin, there are no animal reservoirs for measles. Measles is the most contagious human virus and has co-existed with us for hundreds, if not thousands, of years. Nonimmune individuals exposed to measles have a 90% likelihood of infection. The term *measles* was coined in 1693 by Thomas Sydenham, from the medieval English *mesles* and the Latin *misella*, a diminutive of misery [2]. Measles was endemic in much of Europe, Asia, India, and China since the Middle Ages. Following European colonial expansion in the 16^th^ century, measles spread worldwide with devastating consequences. Severe measles epidemics in Cuba and Honduras reportedly killed nearly 67% of the total native population from 1529-1531 [2,3]. In the wake of a resurgence of measles cases worldwide, it is of interest to note some unique paradoxes of this virus.

## 1. Despite being an RNA virus with an error‑prone polymerase, measles remains antigenically stable over time

The measles vaccine originated in 1954 when a measles outbreak occurred at a boarding school in Massachusetts. Dr. Thomas Peebles and John Franklin Enders successfully cultivated the virus from 11-year-old David Edmonston, termed the “Edmonston-B” strain [4]. The Edmonston-B strain was attenuated by *in vitro* passage in human and chicken cells to generate the measles vaccine which was licensed for public use in 1963 [5]. The vaccine strain is most closely related to genotype A viruses, which are extinct and are not associated with documented endemic transmission in any part of the world [6]. This unique feature allows for distinguishment between natural infection and a potential vaccine-induced adverse event. While the vaccine is a live-attenuated virus, no human-to-human transmission of the measles vaccine strain has ever been documented [7]. Two doses of the measles vaccine confer 97% protection against disease.

Unlike influenza, measles virus only has one serotype or “monotype”, and therefore the antibodies generated in response to infection recognize conserved epitopes across all genotypes [7]. The measles genotypes are determined by the sequence of 450 nucleotides at the end of the nucleoprotein-coding region (termed the N-450 sequence) [8]. In 2003, there were 18 co-circulating genotypes worldwide. This number has since decreased steadily to only 2 genotypes, B3 and D8, since 2021 [8]. In the US and Canada in 2025, the outbreak strain was genotype D8, with only a minority of cases being from genotype B3 [9]. These B3 cases are suspected to stem from separate imports and not due to local transmission, as B3 strains have been circulating in regions of Africa and the Middle East [10]. During this outbreak, the World Health Organization reported that there is “no evidence of decreased vaccine effectiveness or changes in the virus that would result in increased severity” [9].

How is it possible that measles circulates in the global population under selective pressure by the measles vaccine, and yet we don’t detect changes in the virus? Measles is a negative sense RNA virus and requires an RNA-dependent RNA polymerase in order to transcribe and replicate its genome. This process is notoriously error prone, with an estimated error rate of 10^−4^–10^−5^ per site per replication [11]. However, field isolates during outbreaks show very low levels of nucleotide divergence suggesting that measles virus populations do not evolve rapidly [11–13].

What is keeping measles virus from mutating? Measles virus has high genetic stability observed both in laboratory settings and in field isolates. The most variable region of measles virus is the aforementioned N-450 sequence, but even this sequence is stable during outbreaks. One possible contributing factor is the “rule of six” that constrains *paramyxovirinae* family members to a genome length that is a multiple of six [14]. These six nucleotides are the length required to wrap around one nucleoprotein molecule, and this organization would not favor insertion or deletion mutations. *Pneumovirinae*, such as RSV, have similar genetic makeup but are not constrained by the “rule of six”, and these viruses display comparatively high genome variability. Thus, this rule of six may be one possible reason why measles virus exhibits robust genome stability compared to other members of the paramyxovirus family.

In addition to the “rule of six”, experiments performed by Munoz-Alia et al. suggest that measles virus is also constrained by B-cell epitopes on measles virus surface glycoproteins [15]. Hemagglutinin (H), the measles virus glycoprotein responsible for receptor binding, contains seven known antigenic regions, or locations in which antibodies can be made to neutralize virus infectivity. Some of these antigenic regions overlap surfaces necessary for binding viral entry receptors including the epithelial receptor, nectin-4, and the lymphocyte receptor, Signaling Lymphocytic Activation Molecule (SLAM). These measles receptors will be discussed further in paradoxes #2 and #3 below. At least five of these seven sites needed to be disrupted in order to affect the susceptibility of measles virus H to serum neutralization, and therefore conferred no selective advantage to the virus [15]. When these antigenic sites were mutated to escape serum neutralization, the virus lost its ability to bind one of, if not both, receptors [15]. In addition, even if measles virus H is not sensitive to serum neutralization, anti-measles virus Fusion (F) antibodies could still effectively neutralize the virus. However, a virus lacking tropism to SLAM and nectin-4 would prevent pathogenicity and transmission [16,17]. Munoz-Alia and colleagues concluded that “there is a near zero probability for the accidental emergence of a pathogenic measles virus capable of evading vaccine-induced immunity” [15].

To date, there is no evidence of measles vaccine-escape mutants. This is good news, as it supports the continued efficacy of the current vaccine to prevent future measles outbreaks, if widely utilized. The current measles vaccine is given as part of a combined measles-mumps-rubella (MMR) vaccine in a 2-dose series to achieve >95% immunity. The recent resurgence of measles has highlighted the importance of widespread use of vaccination and public health efforts to rebuild confidence in vaccines and increase vaccine adherence.

## 2. Measles virus infection is immunosuppressive but leads to lifelong immunity against itself

Multiple epidemiological studies document that children with a history of measles are more likely to be hospitalized or die from other infectious diseases up to 5 years after clearance of the initial infection [18–20]. Infection by measles virus leads to an induced immunosuppression called immune amnesia, a condition in which the immune system “forgets” previously acquired memory against other pathogens.

Immune amnesia is thought to occur through the targeting of multiple important cell types for the immune system. Recent studies in experimentally infected macaques and observational studies in humans found measles infection resulted in up to a 73% reduction in the preexisting repertoire of pathogen-specific antibodies [21,22]. This reduction took patients 2–3 years to recover protective immunity against non-measles pathogens. There are currently no medical guidelines to help protect patients recovering from immune amnesia, but it has been suggested revaccination with routine childhood vaccines may help mitigate secondary infections.

But how does this occur? One of the primary receptors measles virus utilizes is SLAM (aka CD150), a surface glycoprotein expressed not only on naïve and memory B cells and T cells, but also macrophages, mature dendritic cells, and plasma cells in lymphoid tissues and peripheral blood [23,24]. Measles preferentially infects T and B cells, which leads to subsequent immune-mediated clearance of SLAM+ lymphocyte populations [25,26]. Immune amnesia coincides with lifelong immunity against measles. While preexisting memory T and B cells are depleted, this lymphocyte depletion is masked by a rapid proliferation of new measles-specific lymphocytes [25,27]. These population changes in lymphocyte profiles remain detectable more than a month after recovery from initial infection.

But how do we know protection from measles infection is lifelong and why is this immune response so protective? Measles infection generating lifelong immunity was reported by Peter Ludwig Panum when he observed a measles outbreak on the Faroe Islands in 1846. He noted that individuals who had had measles when the previous 1781 outbreak had occurred on the island, were exempt from measles infection during this 1846 outbreak, even though 65 years had passed [28].

Measles infection leads to a durable immune response though multiple potential mechanisms occurring both during and in the months following infection. Because SLAM^+^ antigen-presenting cells are directly and productively infected, this may play a role in antigen presentation and B cell maturation. Infection of these antigen-presenting cells could lead to robust priming of the innate immune response, which would in turn lead to generation of high affinity measles-specific B cells. Further experimental evidence is needed to validate this hypothesis.

Measles virus-infected lymphocytes and dendritic cells can induce apoptosis. Furthermore, virus-infected monocytes can also induce apoptosis in neighboring uninfected lymphocytes by upregulation of apoptosis associated molecules, such as CD95 and TNF-related apoptosis-inducing ligand-receptor (TRAIL-R) [29–31]. Together, these processes likely contribute to the lymphopenia and immunosuppression observed during infection, however apoptosis alone does not account for all aspects of lymphocyte impairment during immune amnesia.

Another feature of measles that may contribute to robust protection is that viral RNA stays in the blood and tissues weeks to months after clearance of the infectious virus [32–34]. As a result, the immune system remains active for months. Both CD4 and CD8 T cells are needed to clear the virus, but both T cells and antibodies are necessary to reduce viral RNA [35]. A cellular response is necessary for initial virus control, and a humoral response is necessary for clearance of viral RNA and suppression of recurrent infectious virus replication/production. It is speculated that lifelong protective immunity after primary measles virus infection could be a consequence of prolonged antigen stimulation [33,36]. Immune activation and lymphocyte proliferation are evident for months after resolution of rash, and during this period, there is a shift in cytokine production towards promoting B cell proliferation and production of antibody-secreting cells [34].

But what about the vaccine? The measles vaccine induces both antibody and cellular immune responses. Protection is best correlated with the quality and quantity of neutralizing antibodies, primarily against H but with some contribution against F [34]. Antibodies alone protect from disease/rash but not from infection. T cells alone do not protect from infection or disease but facilitate clearance of viral RNA [35,37]. Over time antibodies levels and CD4 T cells decrease leading to a vaccine failure rate of ~5% [37–39].

It is important to note that unlike naturally acquired measles immunity, the measles vaccine does not result in immune amnesia [22]. By preserving the immunity of children infected within the first 10 years of life, the measles vaccine may have drastically lowered the baseline childhood morbidity and mortality rate [18].

## 3. Measles is a respiratory virus where the respiratory epithelia are not the initial site of infection

**Fig 1:**
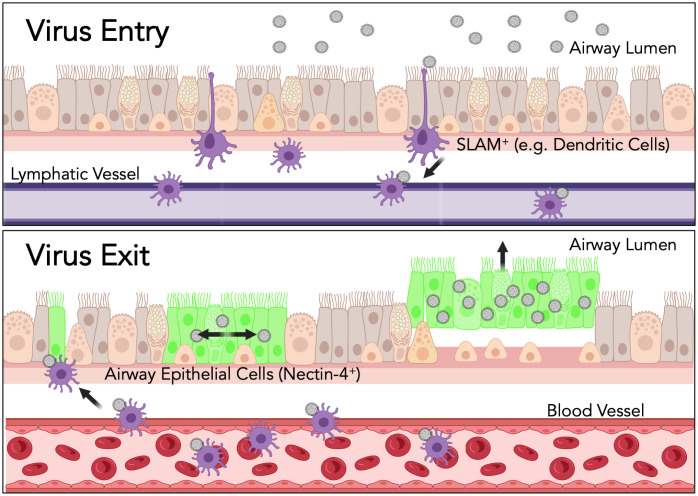
Graphical Summary of Paradox #3. Measles virus enters the host via SLAM^+^ lymphocytes and myeloid cells, such as dendritic cells or pulmonary macrophages, then travels via lymphatic vessels for systemic spread. Measles virus is potentially returned back to the respiratory tract via migratory lymphocytes, macrophages, or dendritic cells traveling in blood vessels (for simplicity, only dendritic cells are depicted in the figure). SLAM^+^ cells bring virus to interact with nectin-4+ airway epithelial cells from the basolateral side. Virus then spreads laterally until infected cells are shed into the lumen to be coughed out, leading to transmission. Created in BioRender. McCray, P. (2026). https://BioRender.com/vu62axw.

We know of measles as a respiratory virus due to the obvious respiratory symptoms and transmission. Often, a respiratory virus enters a host via the upper respiratory system and exits via that same respiratory route. Previously, Fields Virology 1^st^–5^th^ editions cited that the “initial infection [of measles virus] is established in the respiratory tract” [40,41]. As a result of a series of studies, this paradigm was rewritten in the 6^th^ edition [42]. It is now accepted that measles instead infects tissue resident SLAM^+^ lymphocytes, dendritic cells, or macrophages in the respiratory epithelia first, these migratory SLAM^+^ cells travel to the lymph nodes, closely followed by systemic spread (Fig 1, upper panel) [42]. This systemic spread occurs during the prodromal phase, or period before the appearance of the characteristic measles rash. Finally, the virus is brought back to the respiratory epithelia via lymphocytes, macrophages, and/or dendritic cells, leading to infection and finally viral shedding (Fig 1, lower panel) [43–46]. Interestingly, systemic infection of the immune cells occurs asymptomatically. The diagnostic onset of rash and respiratory symptoms instead coincides with return of virus to the epithelium.

The epithelial receptor to measles is nectin-4 (aka PVRL4) [47,48]. Nectin-4 is an adherens junction protein, functioning to maintain the structural integrity between airway epithelia. Infection in the lung is mediated by nectin-4, which is located at the basolateral side of airway epithelial cells [49]. Infection from the apical side facing the lumen of the lung is prevented by tight junctions between cells that restrict receptor access to the virus [49]. However, the receptor is available from the basolateral side, further supporting the model that infection of the lung occurs subsequent to infected migratory lymphocytes moving to the lungs.

Once an airway cell is infected, measles virus spreads directly from one infected cell to its uninfected adjacent neighboring cell [50]. This process continues to form an infectious center which can include anywhere from 30 to 100+ cells. In primary human airway epithelia cells, these infectious centers are able to detach as a single unit and the majority of virus is cell-associated within these infectious centers, as opposed to free virus on the cell surface or in the basolateral media [46]. Observational evidence of infectious centers has been documented from macaque infections, where multinucleated cells or infection associated lesions are present in the epithelium [26,45]. High levels of epithelial cell infection and epithelial damage due to shedding of infectious centers into the lumen may instigate coughing and sneezing, leading to virus transmission. These transmission events may include the release of both free measles virus as well as measles-infected cells or cellular debris.

Further evidence supporting the lung as the final site of infection was highlighted by a study in which rhesus macaques were experimentally infected with a nectin-4-blind measles virus. The resulting symptoms did not include any respiratory symptoms. Instead, macaques only exhibited malaise and skin rash, known symptoms of systemic viral infection. In addition, the humoral immune response resembled wild-type measles virus-infected animals and the macaques still produced strong neutralizing antibodies and controlled viral replication [16]. However, there was no detectable shed virus from the airways. Conversely, when macaques were infected by a SLAM-blind measles virus, there were no clinical signs of infection, including skin rash or anorexia [17]. Taken together, these experiments are consistent with the updated paradigm that infection of airway epithelial cells is not necessary for initiation of systemic infection, however it is necessary for host-to-host viral transmission.

## Conclusion

In summary, measles virus offers a series of a seemingly self-contradictory observations that prove to be well founded. These paradoxes challenge assumptions about viral evolution, immunity, and pathogenesis. Despite being an RNA virus with an inherently error‑prone polymerase, measles remains antigenically stable, constrained by the structural and functional requirements of its glycoproteins. Although natural infection induces profound immune amnesia, it generates lifelong immunity against itself. While measles is transmitted as a respiratory pathogen, its replication strategy places the airway epithelium at the end, rather than the beginning, of the infection cycle. Together, these features underscore the extraordinary evolutionary balance measles virus has achieved over centuries of coexistence with humans. While this review highlights some potential explanations for these paradoxes, there are still many open questions regarding measles biology and pathogenesis. Understanding these biological contradictions not only provides insight into measles pathobiology but also highlights the necessity of widespread vaccination in preventing both acute disease and its long‑term immunological consequences.

## References

[1] Düx A, Lequime S, Patrono LV, Vrancken B, Boral S, Gogarten JF, et al. Measles virus and rinderpest virus divergence dated to the sixth century BCE. Science. 2020;368(6497):1367–70. doi: 10.1126/science.aba9411 32554594 PMC7713999

[2] Berche P. History of measles. Presse Med. 2022;51(3):104149. doi: 10.1016/j.lpm.2022.104149 36414136

[3] Cook SF. The Incidence and significance of disease among the aztecs and related tribes. Hispanic American Historical Review. 1946;26(3):320–35. doi: 10.1215/00182168-26.3.320

[4] Enders JF, Peebles TC. Propagation in tissue cultures of cytopathogenic agents from patients with measles. Proc Soc Exp Biol Med. 1954;86(2):277–86. doi: 10.3181/00379727-86-21073 13177653

[5] Katz SL, Enders JF, Holloway A. The development and evaluation of an attenuated measles virus vaccine. Am J Public Health Nations Health. 1962;52(2)Suppl(Suppl 2):5–10. doi: 10.2105/ajph.52.suppl_2.5 14454407 PMC1522581

[6] Rota PA, Brown K, Mankertz A, Santibanez S, Shulga S, Muller CP, et al. Global distribution of measles genotypes and measles molecular epidemiology. J Infect Dis. 2011;204 Suppl 1:S514-23. doi: 10.1093/infdis/jir118 21666208

[7] Greenwood KP, Hafiz R, Ware RS, Lambert SB. A systematic review of human-to-human transmission of measles vaccine virus. Vaccine. 2016;34(23):2531–6. doi: 10.1016/j.vaccine.2016.03.092 27083423

[8] Bankamp B, Kim G, Hart D, Beck A, Ben Mamou M, Penedos A, et al. Global update on measles molecular epidemiology. Vaccines (Basel). 2024;12(7). Epub 20240722. doi: 10.3390/vaccines12070810 ; PMCID: PMC11281501 39066448 PMC11281501

[9] WHO. Disease outbreak news; measles in the United States of America. 2025.

[10] Namuwulya P, Turyahabwe I, Nakyeyune R, Birungi M, Tushabe P, Eliku JP, et al. Emergence of measles virus genotype D8 amidst endemic B3 circulation in Uganda, 2023-2025. Int J Infect Dis. 2026;163:108255. doi: 10.1016/j.ijid.2025.108255 41319789

[11] Schrag SJ, Rota PA, Bellini WJ. Spontaneous mutation rate of measles virus: direct estimation based on mutations conferring monoclonal antibody resistance. J Virol. 1999;73(1):51–4. doi: 10.1128/JVI.73.1.51-54.1999 9847306 PMC103807

[12] Rota JS, Hummel KB, Rota PA, Bellini WJ. Genetic variability of the glycoprotein genes of current wild-type measles isolates. Virology. 1992;188(1):135–42. doi: 10.1016/0042-6822(92)90742-8 1566568

[13] Rima BK, Earle JA, Baczko K, ter Meulen V, Liebert UG, Carstens C, et al. Sequence divergence of measles virus haemagglutinin during natural evolution and adaptation to cell culture. J Gen Virol. 1997;78 (Pt 1):97–106. doi: 10.1099/0022-1317-78-1-97 9010291

[14] Beaty SM, Lee B. Constraints on the genetic and antigenic variability of measles virus. Viruses. 2016;8(4):109. doi: 10.3390/v8040109 27110809 PMC4848602

[15] Muñoz-Alía MÁ, Nace RA, Zhang L, Russell SJ. Serotypic evolution of measles virus is constrained by multiple co-dominant B cell epitopes on its surface glycoproteins. Cell Rep Med. 2021;2(4):100225. doi: 10.1016/j.xcrm.2021.100225 33948566 PMC8080110

[16] Leonard VHJ, Sinn PL, Hodge G, Miest T, Devaux P, Oezguen N, et al. Measles virus blind to its epithelial cell receptor remains virulent in rhesus monkeys but cannot cross the airway epithelium and is not shed. J Clin Invest. 2008;118(7):2448–58. doi: 10.1172/JCI35454 18568079 PMC2430500

[17] Leonard VHJ, Hodge G, Reyes-Del Valle J, McChesney MB, Cattaneo R. Measles virus selectively blind to signaling lymphocytic activation molecule (SLAM; CD150) is attenuated and induces strong adaptive immune responses in rhesus monkeys. J Virol. 2010;84(7):3413–20. doi: 10.1128/JVI.02304-09 20071568 PMC2838096

[18] Mina MJ, Metcalf CJE, de Swart RL, Osterhaus ADME, Grenfell BT. Long-term measles-induced immunomodulation increases overall childhood infectious disease mortality. Science. 2015;348(6235):694–9. doi: 10.1126/science.aaa3662 25954009 PMC4823017

[19] Gadroen K, Dodd CN, Masclee GMC, de Ridder MAJ, Weibel D, Mina MJ, et al. Impact and longevity of measles-associated immune suppression: a matched cohort study using data from the THIN general practice database in the UK. BMJ Open. 2018;8(11):e021465. doi: 10.1136/bmjopen-2017-021465 30413497 PMC6231568

[20] Behrens L, Cherry JD, Heininger U, Swiss Measles Immune Amnesia Study Group. The susceptibility to other infectious diseases following measles during a three year observation period in Switzerland. Pediatr Infect Dis J. 2020;39(6):478–82. doi: 10.1097/INF.0000000000002599 32084116

[21] Laksono BM, de Vries RD, Verburgh RJ, Visser EG, de Jong A, Fraaij PLA, et al. Studies into the mechanism of measles-associated immune suppression during a measles outbreak in the Netherlands. Nat Commun. 2018;9(1):4944. doi: 10.1038/s41467-018-07515-0 30470742 PMC6251901

[22] Mina MJ, Kula T, Leng Y, Li M, de Vries RD, Knip M, et al. Measles virus infection diminishes preexisting antibodies that offer protection from other pathogens. Science. 2019;366(6465):599–606. doi: 10.1126/science.aay6485 31672891 PMC8590458

[23] Tatsuo H, Ono N, Tanaka K, Yanagi Y. SLAM (CDw150) is a cellular receptor for measles virus. Nature. 2000;406(6798):893–7. doi: 10.1038/35022579 10972291

[24] De Salort J, Sintes J, Llinàs L, Matesanz-Isabel J, Engel P. Expression of SLAM (CD150) cell-surface receptors on human B-cell subsets: from pro-B to plasma cells. Immunol Lett. 2011;134(2):129–36. doi: 10.1016/j.imlet.2010.09.021 20933013

[25] de Vries RD, McQuaid S, van Amerongen G, Yüksel S, Verburgh RJ, Osterhaus ADME, et al. Measles immune suppression: lessons from the macaque model. PLoS Pathog. 2012;8(8):e1002885. doi: 10.1371/journal.ppat.1002885 22952446 PMC3431343

[26] de Swart RL, Ludlow M, de Witte L, Yanagi Y, van Amerongen G, McQuaid S, et al. Predominant infection of CD150+ lymphocytes and dendritic cells during measles virus infection of macaques. PLoS Pathog. 2007;3(11):e178. doi: 10.1371/journal.ppat.0030178 18020706 PMC2077902

[27] Petrova VN, Sawatsky B, Han AX, Laksono BM, Walz L, Parker E, et al. Incomplete genetic reconstitution of B cell pools contributes to prolonged immunosuppression after measles. Sci Immunol. 2019;4(41):eaay6125. doi: 10.1126/sciimmunol.aay6125 31672862

[28] Emerson H. Panum on measles: observations made during the epidemic of measles on the Faroe Islands in the year 1846. Am J Public Health. 1940;30(10):1245–6.

[29] Griffin DE. Measles immunity and immunosuppression. Curr Opin Virol. 2021;46:9–14. doi: 10.1016/j.coviro.2020.08.002 32891958 PMC7994291

[30] Fugier-Vivier I, Servet-Delprat C, Rivailler P, Rissoan MC, Liu YJ, Rabourdin-Combe C. Measles virus suppresses cell-mediated immunity by interfering with the survival and functions of dendritic and T cells. J Exp Med. 1997;186(6):813–23. doi: 10.1084/jem.186.6.813 9294136 PMC2199042

[31] Okada H, Kobune F, Sato TA, Kohama T, Takeuchi Y, Abe T, et al. Extensive lymphopenia due to apoptosis of uninfected lymphocytes in acute measles patients. Arch Virol. 2000;145(5):905–20. doi: 10.1007/s007050050683 10881678

[32] Riddell MA, Moss WJ, Hauer D, Monze M, Griffin DE. Slow clearance of measles virus RNA after acute infection. J Clin Virol. 2007;39(4):312–7. doi: 10.1016/j.jcv.2007.05.006 17625962

[33] Lin W-HW, Kouyos RD, Adams RJ, Grenfell BT, Griffin DE. Prolonged persistence of measles virus RNA is characteristic of primary infection dynamics. Proc Natl Acad Sci U S A. 2012;109(37):14989–94. doi: 10.1073/pnas.1211138109 22872860 PMC3443140

[34] Griffin DE. The immune response in measles: virus control, clearance and protective immunity. Viruses. 2016;8(10). doi: 10.3390/v8100282 27754341 PMC5086614

[35] Lin WH, Pan CH, Adams RJ, Laube BL, Griffin DE. Vaccine-induced measles virus-specific T cells do not prevent infection or disease but facilitate subsequent clearance of viral RNA. mBio. 2014;5(2):e01047. doi: 10.1128/mBio.01047-14 PMC3993862 24736226

[36] Nelson AN, Lin W-HW, Shivakoti R, Putnam NE, Mangus L, Adams RJ, et al. Association of persistent wild-type measles virus RNA with long-term humoral immunity in rhesus macaques. JCI Insight. 2020;5(3):e134992. doi: 10.1172/jci.insight.134992 31935196 PMC7098782

[37] Griffin DE. Measles vaccine. Viral Immunology. 2018;31(2):86–95. doi: 10.1089/vim.2017.0143 29256824 PMC5863094

[38] Naniche D, Garenne M, Rae C, Manchester M, Buchta R, Brodine SK, et al. Decrease in measles virus-specific CD4 T cell memory in vaccinated subjects. J Infect Dis. 2004;190(8):1387–95. doi: 10.1086/424571 15378430

[39] Kontio M, Jokinen S, Paunio M, Peltola H, Davidkin I. Waning antibody levels and avidity: implications for MMR vaccine-induced protection. J Infect Dis. 2012;206(10):1542–8. doi: 10.1093/infdis/jis568 22966129

[40] Fields BN, Knipe DM, Howley PM. Fields virology. 3rd ed. Philadelphia: Lippincott-Raven Publishers; 1996.

[41] Fields BN, Knipe DM, Howley PM. Fields virology. 5th ed. Philadelphia: Wolters Kluwer Health/Lippincott Williams & Wilkins; 2007.

[42] Knipe DM, Howley PM. Fields virology. 6th ed. Philadelphia, PA: Wolters Kluwer/Lippincott Williams & Wilkins Health; 2013.

[43] Frenzke M, Sawatsky B, Wong XX, Delpeut S, Mateo M, Cattaneo R, et al. Nectin-4-dependent measles virus spread to the cynomolgus monkey tracheal epithelium: role of infected immune cells infiltrating the lamina propria. J Virol. 2013;87(5):2526–34. doi: 10.1128/JVI.03037-12 23255790 PMC3571369

[44] Singh BK, Li N, Mark AC, Mateo M, Cattaneo R, Sinn PL. Cell-to-cell contact and nectin-4 govern spread of measles virus from primary human myeloid cells to primary human airway epithelial cells. J Virol. 2016;90(15):6808–17. doi: 10.1128/JVI.00266-16 27194761 PMC4944272

[45] Ludlow M, Lemon K, de Vries RD, McQuaid S, Millar EL, van Amerongen G, et al. Measles virus infection of epithelial cells in the macaque upper respiratory tract is mediated by subepithelial immune cells. J Virol. 2013;87(7):4033–42. doi: 10.1128/JVI.03258-12 23365435 PMC3624209

[46] Hippee CE, Singh BK, Thurman AL, Cooney AL, Pezzulo AA, Cattaneo R, et al. Measles virus exits human airway epithelia within dislodged metabolically active infectious centers. PLoS Pathog. 2021;17(8):e1009458. doi: 10.1371/journal.ppat.1009458 34383863 PMC8384213

[47] Mühlebach MD, Mateo M, Sinn PL, Prüfer S, Uhlig KM, Leonard VHJ, et al. Adherens junction protein nectin-4 is the epithelial receptor for measles virus. Nature. 2011;480(7378):530–3. doi: 10.1038/nature10639 22048310 PMC3245798

[48] Noyce RS, Bondre DG, Ha MN, Lin L-T, Sisson G, Tsao M-S, et al. Tumor cell marker PVRL4 (nectin 4) is an epithelial cell receptor for measles virus. PLoS Pathog. 2011;7(8):e1002240. doi: 10.1371/journal.ppat.1002240 21901103 PMC3161989

[49] Sinn PL, Williams G, Vongpunsawad S, Cattaneo R, McCray PB Jr. Measles virus preferentially transduces the basolateral surface of well-differentiated human airway epithelia. J Virol. 2002;76(5):2403–9. doi: 10.1128/jvi.76.5.2403-2409.2002 11836418 PMC153805

[50] Singh BK, Hornick AL, Krishnamurthy S, Locke AC, Mendoza CA, Mateo M, et al. The nectin-4/afadin protein complex and intercellular membrane pores contribute to rapid spread of measles virus in primary human airway epithelia. J Virol. 2015;89(14):7089–96. doi: 10.1128/JVI.00821-15 25926640 PMC4473566

